# Pre-validation of a novel reconstructed skin equivalent model for skin irritation and nanoparticle risk assessment

**DOI:** 10.1039/d4na00804a

**Published:** 2025-01-08

**Authors:** Priscila Laviola Sanches, Rosana Bizon Vieira Carias, Gutember Gomes Alves, Carolina Motter Catarino, Bruna Bosquetti, Meg Cristina De Castilho Costa, Andrezza Di Pietro Micali, Desirée Cigaran Schuck, José Mauro Granjeiro, Ana R. Ribeiro

**Affiliations:** a Postgraduate Program in Translational Biomedicine, University of Grande Rio Duque de Caxias Brazil jmgranjeiro@gmail.com; b Directorate of Scientific, Industrial and Technology Metrology, National Institute of Metrology, Quality and Technology Duque de Caxias Brazil; c Regenerative Medicine Laboratory, Faculdade de Medicina de Petrópolis UNIFASE/FMP Rio de Janeiro Brazil; d Department of Molecular and Cell Biology, Institute of Biology, Fluminense Federal University Niterói Rio de Janeiro Brazil; e Clinical Research Unit, Antonio Pedro Hospital, Fluminense Federal University Niterói Brazil; f Product Safety Management, Grupo Boticário São José dos Pinhais Paraná Brazil carolina.catarino@grupoboticario.com.br; g School of Dentistry, Fluminense Federal University Niterói Brazil; h Nanosafety Group, International Iberian Nanotechnology Laboratory Braga Portugal ana.ribeiro@inl.int

## Abstract

In alignment with the global movement toward reducing animal testing, several reconstructed human epidermis (RHE) models have been created for conducting skin irritation tests. These models have undergone development, verification, validation, and integration into OECD TG 439. Our team has introduced a novel in-house RHE named GB-RHE, and we adhere to OECD TG 439 to pre-validate the model and test its potential employment for nanoparticle irritation studies. GB-RHE exhibits morphological, biochemical, and physiological attributes equivalent to the human epidermis, featuring well-differentiated multilayered viable keratinocytes with a robust barrier function. The performance of the GB-RHE model was evaluated using ten reference chemicals, following the performance standard of OECD TG 439. The results demonstrated commendable predictive capacity and showed that titanium dioxide nanoparticles (TiO_2_ NPs) are ‘non-irritant’ to the human epidermis following the globally harmonized classification system. However, although the histological analysis did not show morphological changes, transmission electron micrographs demonstrated that TiO_2_ NPs can be internalized, reaching the external viable layers of the epidermis. This study demonstrates that in addition to the potential of the GB-RHE model to evaluate skin irritation, this model also has the potential to evaluate the skin toxicity of NPs and carry out cell internalization studies.

## Abstract 2

Schematic of reconstructed epidermis model, with and without nanoparticle exposure.

## Graphical abstract

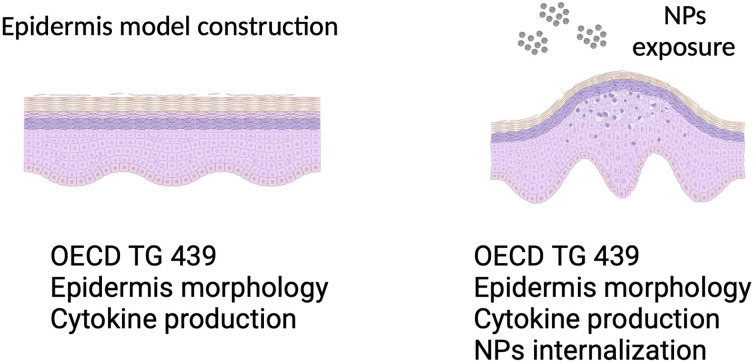

## 1. Introduction

Skin equivalent models have emerged as crucial *in vitro* alternatives to animal testing, widely utilized for assessing the safety and efficacy of substances, including chemicals, drugs, and cosmetics. These models are meticulously designed to mimic the skin's mechanical, histological, and biological properties, such as elasticity, thickness, and permeability.^1,2^ Various types of skin models exist, ranging from the simpler reconstructed human epidermis (RHE), which includes only the epidermis, to more complex full-thickness (FT) models that encompass both the epidermis and dermis, each offering distinct advantages and limitations.^3,4^

Among these, the RHE model is predominantly employed. It is composed of human keratinocytes grown in a collagen matrix or other extracellular matrix-based scaffold that closely recapitulates the *in vivo* environment of the epidermis.^5,6^ To achieve physiological relevance, an air–liquid interface is essential for complete skin differentiation and cornification across the model's surface.^7^ For instance, the Episkin™ model utilizes human keratinocytes grown for 13 days in a collagen matrix at the air–liquid interface, whereas in the SkinEthic™ model, human keratinocytes are seeded on a polycarbonate filter for 17 days in the air–liquid interface.^8^

RHE models have been effective in assessing the safety and efficacy of topically applied substances',^9^ predicting irritation with an accuracy rate of up to 93%.^10,11^ Beyond skin irritation, these models are valuable in permeation studies, showing a high correlation with *in vivo* data, and they are also used in researching skin sensitization, aging mechanisms, the effectiveness of anti-aging compounds, as well as inflammation and wound healing.^12–14^ For example, Tancos *et al.* demonstrated that RHE models could be used to study the influence of environmental pollutants on skin health, revealing significant gene expression changes associated with skin aging caused by particulate matter.^15^ The currently available RHE models have been demonstrated to be a reliable and cost-effective alternative to animal testing.

Recent advancements in nanotechnology, particularly the use of nanoparticles (NPs), have led to concern about possible deleterious human exposure and potential adverse effects on human health. In cosmetics, NPs enhance product efficacy, texture, and stability. Nanoparticle mechanisms of toxicity are related to the formation of reactive oxygen species, genotoxicity, and inflammatory responses, among others.^16^ However, for novel formulations, their possible hazardous effect on the skin, which includes irritation, sensitization, and corrosion, still needs to be assessed. In this way, evaluating the safety of NPs employed in cosmetics using equivalent models is a subject of ongoing research.

Currently, whether skin-equivalent models are appropriate for assessing the cytotoxicity, corrosion, irritation, and phototoxicity of NP-containing formulations is uncertain. The evaluation principles for chemical product risk assessment are often applied to NPs. However, it should be stressed that NPs' physicochemical characteristics (size, reactivity, surface area, concentration, crystalline structure, among others) are considered determinants for their safety assessment. Given the absence of comprehensive regulatory frameworks, assessing NP risks involves case-by-case considerations.^17–19^

Notably, due to their photoprotective capacity, TiO_2_ NPs are frequently utilized in sunscreens.^20^ These TiO_2_ NPs offer transparency to visible light and better UV-blocking efficiency compared to micrometre-sized sunscreen components known to be opaque. TiO_2_ NPs reflect and scatter UVB (290–320 nm) and UVA (320–400 nm), providing adequate solar radiation protection and, consequently, preventing sunburn and photoaging.^21,22^ Despite being perceived as relatively safe, some studies have reported oxidative stress and inflammation in human keratinocytes upon exposure to TiO_2_ NPs.^23–26^

Addressing these concerns, a survey carried out in the United States estimated daily TiO_2_ NPs exposure from personal care products (toothpaste and sunscreen) at 2.8 to 14 mg per person.^19^ In this study, we developed and pre-validated a novel in-house RHE model called GB-RHE, adhering to the OECD TG 439 protocol to evaluate skin irritation using ten reference chemicals (a subset of substances in line with the validation protocol). Transepithelial electrical resistance (TEER) measurements reflected the model's robust epidermal barrier (comparable to other OECD-validated models), with average values of 1649 ohms cm^2^, and histological analysis confirmed its morphological accuracy. Tested with OECD Performance Standard chemicals, the GB-RHE model demonstrated 100% sensitivity and 60% specificity, with an overall accuracy of 80%. To extend the GB-RHE model's validation, we assessed its capability to evaluate nanoparticles' irritation potential, particularly titanium dioxide nanoparticles (TiO_2_ NPs), following the OECD TG 439 protocol. Results indicated TiO_2_ NPs to be non-irritant, showcasing the GB-RHE model as a reliable methodology for assessing the irritation potential of chemicals and nanoparticles, thus enhancing its applicability in cosmetics and nanotoxicology research.

## 2. Materials and methods

### 2.1 Isolation of human primary keratinocyte and their culture

The keratinocytes were obtained from human foreskins. This study was approved by the Research Ethics Committee of Universidade do Grande Rio Professor José de Souza Herdy (references 46799215.1.0000.5283) in accordance with the Declaration of Helsinki. All study participants signed the informed consent form. Initially, a study was carried out to evaluate the proliferation of primary keratinocytes derived from human skin biopsies from different age groups. Then, the entire study was carried out using foreskins derived from circumcisions of children between 0 and 5 years of age, where the best results concerning cell proliferation were observed. The specimens measuring approximately 1 cm^2^ were cut into 0.3 cm^2^ pieces and were incubated in Dispase II 7.2 U/mL (Gibco; 17105041) solution in phosphate-buffered saline solution (PBS) (Gibco; 20012027) for 16–18 hours at 4 °C. After which the epidermis sheets were manually removed from the dermis and were incubated in 0.05% trypsin (Gibco; 27250018)–0.02% EDTA (Sigma-Aldrich, E6758) solution for 3–5 minutes at 37 °C, under agitation. The enzyme was inactivated by adding a soybean trypsin inhibitor of 1.0 mg mL^−1^ (Sigma-Aldrich L1395). The cell suspension was passed by filtration (70 μm) and collected after centrifugation at 400*g* for 10 minutes. The cells were counted with trypan blue dead cell exclusion dye and plated on cell culture plastic at a density of 20 000 cells per cm^2^ in Keratinocyte Growth Medium KGM-Gold® (LONZA, culture medium, 0000973113; supplement, 0000957331) and incubated in a humidified atmosphere with 5% CO_2_ at 37 °C.

### 2.2 Cells morphological analysis

The initial passage (P0) was screened every day until the colony proliferation cells were big, without direct contact among them. The cells were passed with a triple reagent (Gibco; 12563029), and the enzyme was inactivated by adding soybean trypsin inhibitor, as described above. Sub-culturing was done till P7 when the monolayer reached around 70–80% confluency by seeding at 2000 cells per cm^2^ density. The primary cultures had their morphological appearance monitored through observation and image capture using an inverted optical microscope with phase contrast (Nikon, model TS 100) at all stages of cultivation.

### 2.3 Determination of cell proliferation rate

At each subculture process, cells were quantified, and the overall number recovered was used in correlation with the number of live cells seeded in the late process to calculate the duplication level.

### 2.4 Construction of the Grupo Boticário reconstructed human epidermis model (GB-RHE)

The RHE model was produced using primary keratinocytes provided by the Faculty of Medicine of Petrópolis/UNIFASE, coded as hKT MB 04 and hKT MB 32. All cells were placed in vials for freezing and kept in liquid nitrogen. After thawing, cells were expanded into 25 and/or 75 cm^2^ cell culture flasks. The medium used to culture the keratinocytes was the KGM culture medium (Keratinocyte Growth Medium, Lonza, lot 0000973113) supplemented with keratinocyte growth factors (lot 000097331). After being seeded, the cells were kept in a humidified incubator containing 95% air and 5% CO_2_ at 37 °C.

To build the GB-RHE model, 24-well plates with inserts were used (Greiner Bio-one, lot 21200116). Before adding the cells, all inserts were coated with 40 μL of a 0.03 mg mL^−1^ collagen IV solution in serum-free DMEM for 2 hours; then, the inserts were washed with DMEM with 10% fetal bovine serum (FBS). 150 000 cells in 100 media were seeded per insert, and 500 μL of KGM medium was added to the bottom of the insert (in the well of the plate). After 48 hours, the KGM medium was removed, and the differentiation medium was added. Every two days, the differentiation medium was changed until the desired differentiation period was completed (8 to 11 days of differentiation were evaluated).

All monolayer cell morphology images were obtained in an inverted optical microscope (Nikon Eclipse TS100) using the imaging program (Leica Applications Suites – LAS EZ). The histology images of the skin model were obtained in an inverted optical microscope from Zeiss Axiovert, using the Zeiss AxionVision program.

For histological analyses, the tissues were fixed with 4% formaldehyde and embedded in paraffin, and the slides were stained with hematoxylin and eosin.

In parallel, Grupo Boticário also generated the reconstructed model in-house to perform an interlaboratory validation study for the skin irritation potential protocol described in the next section. Human primary keratinocytes were purchased from Gibco (HEKn, 2437268) and cultivated under equivalent conditions.

### 2.5 Irritation test according to OECD TG 439

The OECD 439 protocol: *In vitro* skin irritation: reconstructed human epidermis test method, 2019, was used as the basis for performing the skin irritation test. The irritation test used the 10 chemical substances described by OECD TG 439. The information for each chemical substance (all from Sigma) is found in Table 1. Only deionized water was added to the untreated skins. As a positive control, sodium dodecyl sulfate (SDS, Sigma, lot SLCD7856) was used, and as a negative control, PBS, and the skin without treatment. A fixed volume of 40 μL was used for liquid substances, and for solid substances, 10 mg was used on the surface of each reconstructed skin, previously moistened with 40 μL of deionized water. In untreated skins, only deionized water was added. After 42 minutes of exposure, the skins were carefully washed with at least 20 mL of PBS. The PBS was removed entirely with a pipette, the bottom skin medium was changed, and the skins were incubated for another 42 hours.

**Table 1:** Proficiency substances following the OECD TG 439^a^

	Substance	CAS	Category	State	Lot
1	Naphthalene acetic acid	86-87-3	Non-irritant	Solid	SLBR4706V
2	Isopropanol	67-63-0	Non-irritant	Liquid	K50189835
3	Methyl stearate	112-61-8	Non-irritant	Solid	BCBZ9969
4	Heptyl butyrate	5870-93-9	Non-irritant	Liquid	SHBM5081
5	Hexyl salicylate	6259-76-3	Non-irritant	Liquid	BCBZ4977
6	Cyclamen aldehyde	103-95-7	Irritant	Liquid	SHBL7554
7	1-Bromohexane	111-25-1	Irritant	Liquid	WXBC3558V
8	Potassium hydroxide (5% aq.)	1310-58-3	Irritant	Liquid	STBJ6441
9	1-Methyl-3-phenyl-1-piperazine	5271-27-2	Irritant	Solid	MKCK3453
10	Heptanal	111-71-7	Irritant	Liquid	STBB1907V

a Subset from validation reference substances list.

Cell viability was evaluated with the MTT indicator (thiazolyl blue tetrazolium bromide – Sigma, lot MKCF0652). The MTT solution (500 μL) at a concentration of 1 mg mL^−1^ was added to the bottom of each well and kept in contact with the skins for 3 hours, without light, at 37 °C and 5% CO_2_. After incubation, the epidermis was immediately placed in isopropanol (Merck) for 16–18 hours under refrigeration. The absorbance reading was measured at 570 nm using a spectrophotometer (SoftMax Pro 5.4). The viability for each sample tested was calculated relative to the untreated control. The substance was considered non-irritating if cell viability compared to the negative control was ≥50%, and the substance was considered irritating if cell viability compared to the control was <50%.

### 2.6 Evaluation of TiO_2_ NP interference with the MTT assay

We used a water-killed RHE for irritation testing to assess TiO_2_ NP interference in the MTT assay, following the modified DB-ALM Protocol #135: SkinEthic™ Skin Irritation Test. For this, live epidermis was added to a 24-well plate containing 300 μL of distilled water and incubated at 37 °C, 5 °C of CO_2_, for 24 ± 1 h. At the end of the incubation, the water was discarded, and the dead epidermis was frozen at −20 °C. On the day of the irritation test, tissues were thawed at room temperature for 10 minutes in the maintenance medium. Next, 40 μL of different concentrations of TiO_2_ NPs (0 μg mL^−1^ (control), 10 μg mL^−1^, and 100 μg mL^−1^) were applied in the RHE samples for 48 hours. After this time, the skins were carefully washed with at least 20 mL of PBS. The PBS was removed entirely with the aid of the pipette, the bottom skin medium was changed, and the skins were incubated for another 42 hours. This study was performed on triplicate skin. Cell viability was evaluated with the MTT assay described in the previous section.

### 2.7 Analysis of the irritation and cytotoxicity potential of TiO_2_ NPs on the GB-RHE

Irritation and cytotoxicity tests were performed using two different concentrations of TiO_2_ NPs (10 μg mL^−1^ and 100 μg mL^−1^). As a positive control, SDS was used, and as a negative control, PBS and the skin without treatment. A volume of 40 μL of each sample was added to the surface of the RHE sample. Only deionized water was added to the untreated skins. The nanoparticle irritation was assessed with a 42 minutes exposure, while cytotoxicity analysis was performed during a 48 hours exposure period, tailoring the duration to suit each specific test requirement. After these times, the skins were carefully washed with at least 20 mL of PBS. To assess irritation, the PBS was removed entirely with the aid of the pipette, the bottom skin medium was changed, and the skins were incubated for another 42 hours. This study was carried out using triplicate skin. Cell viability was evaluated with the MTT indicator, as described in the previous section.

### 2.8 Detection of cytokines and growth factors

The determination of the concentration of cytokines and growth factors secreted after exposure of GB-RHE to the 10 chemicals used in the irritation test, and after exposure to different concentrations of NPs was carried out through the multiparametric test using XMap Luminex magnetic microsphere. Quantification of interleukin (IL) IL-6, IFN-gamma, IL-1RA, GM-CSF, G-CSF, TNF-alpha, RANTES, Eotaxin, FGF2, VEGF, PDGF, IP-10, MCP-1, MIP-1a, MIP-1b, IL-1beta, IL-2, IL-5, IL-7, IL-8, IL-9, IL-10, IL-15, IL-12 (p70) and IL-17 A, was performed using a commercial kit (27 Plex panel, BioRad, California, USA). A BioPlex MAGPIX system (BioRad) was employed to quantify the magnetic beads and results were analyzed using Xponent v software 3.0 (Luminexcorp, USA).

### 2.9 Titanium dioxide nanoparticles internalization on GB-RHE

**Fig. 1:**
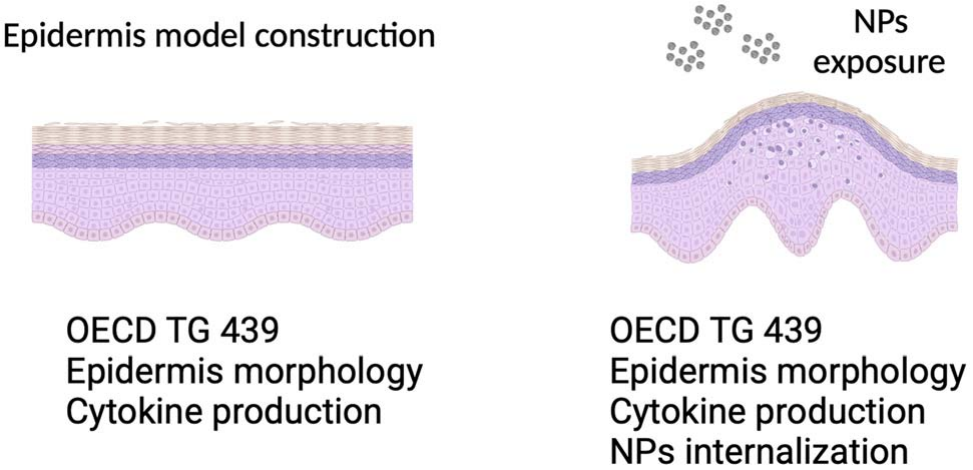
Schematic of reconstructed epidermis model, with and without nanoparticle exposure.

After 48 hours of exposure to different concentrations of TiO_2_ NPs (control (0 μg mL^−1^), 10 μg mL^−1^, and 100 μg mL^−1^), the skins were washed with 0.1 M cacodylate buffer and perforated into sizes of approximately 1 mm^3^. They were then fixed in 2.5% (v/v) glutaraldehyde, post-fixed for 1 hour in 1% OsO_4_ (osmium tetroxide), and potassium ferritin 0.8% (1 : 1), dehydrated in acetone and included in Epon. The ultrathin sections were counterstained with 10% uranyl acetate and 5% lead citrate and observed under a transmission electron microscope (Tecnai Spirit G2, FEI, Eindhoven, Netherlands). A resume of the experimental setup is presented in Fig. 1.

## 3. Results

### 3.1 Characterization of human primary keratinocyte culture and their contribution to constructing an RHE model with desired biological characteristics

**Fig. 2:**
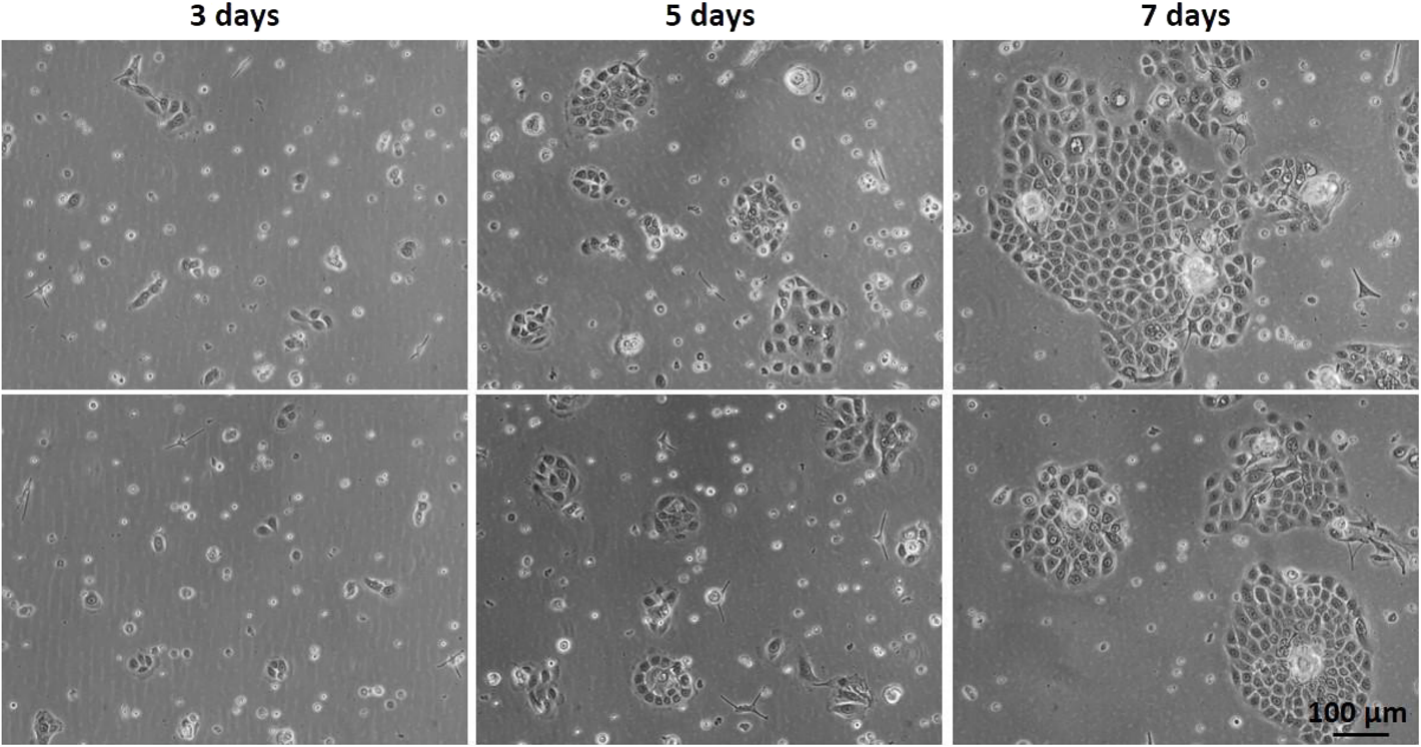
Optical microscopy of primary keratinocyte culture after initial plating (#0) on different days of culture: 3 days, 5 days, and 7 days.

The isolation of keratinocytes from the foreskin epidermis yields cells that maintain their epithelial characteristics of staying closely connected. They proliferate into colonies with well-defined edges, as depicted in Fig. 2. These features are crucial for cultivating a quality culture of epithelial progenitor cells, which is essential for successfully constructing reconstructed skin.

**Fig. 3:**
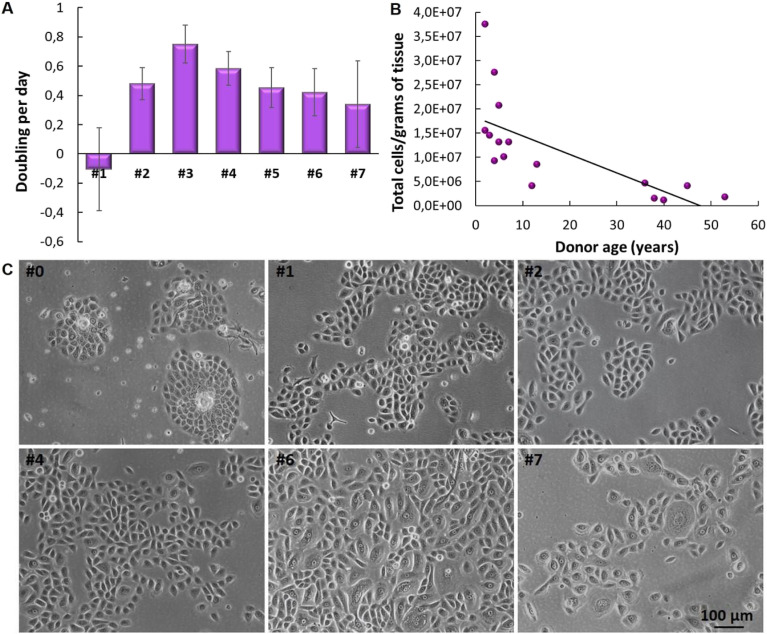
Cultivation of human primary keratinocytes cultured *in vitro*. (A) Graph of proliferation with different cell passages. (B) Graph depicting the correlation between the number of primary keratinocytes derived from human skin biopsies and the donors' age. (C) Optical microscopy images of cultures at different cell passages.

Human primary keratinocytes derived from the foreskin epidermis exhibited a limited proliferation period *in vitro*. The proliferation curve shows a peak at passage 3 (#3) after which proliferation declines (Fig. 3A). Before initiating the construction of 3D models, it is crucial to evaluate the donor's age, as samples from older donors display a reduced proliferative rate and lower yield per gram of tissue processed (Fig. 3B). As already reported in literature, optical microscopy images reveal that, over time in culture, keratinocytes enlarge and spread more evenly on the surface, losing their colony formation characteristic. This transition indicates a shift from epithelial progenitor cells to mature epithelial cells (Fig. 3C). In passages #0, #1, #2 and #4, predominantly young and proliferative cultures are observed, with cuboidal/fusiform morphology and small cytoplasm; in #6, cells with mixed morphology are observed, small and fusiform or large and rounded, and in #7 a smaller number of cells indicating a decrease in their proliferation rate with cells demonstrating senescent characteristics (such as increased vacuolated cytoplasm).

**Fig. 4:**
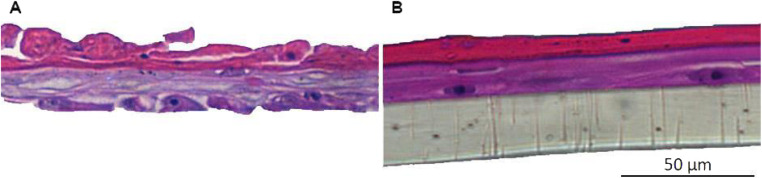
Histology of unsuccessful skin model reconstruction using different cell batches. (A) Lot 1 and (B) lot 2.

Cultivating primary keratinocytes requires meticulous handling and attention. For instance, during cell culture, it is important to prevent the keratinocytes from reaching full confluence, as this can lead to differentiation or senescence. This is identifiable through morphological characteristics such as the increase in cell size (volume), highly vacuolated, and polynucleated, leading to a detachment of non-senescent cells. To construct an effective RHE model, it is essential to use young, proliferative primary keratinocytes. If older cells are used, the resulting skin model tends to have fewer cell layers, compromising epidermis barrier integrity and stratification. Fig. 4 depicts epidermis models constructed with keratinocytes that lack appropriate morphological features. Consequently, these models exhibit limited average thickness (20.5 μm and 22.14 μm, respectively). These variations are attributed to cell batches, particularly regarding passage numbers and donor characteristics. The incomplete formation of the epidermis, lacking all the necessary layers, is further evidenced by the reduced optical density (OD) value of the negative control in the MTT assay—measured at 0.28 and 0.4 for skin samples from the same experiments shown in Fig. 4A and B, respectively. According to OECD TG 439, there is an acceptability range for negative control OD values in the MTT assay, which may vary from model to model. However, the lowest acceptance value presented in OECD TG439 is an OD of 0.6 for EpiSkin™. These initial results demonstrate the relevance of considering keratinocytes' quality to reconstruct an RHE with the required adequate morphological and biological characteristics.

### 3.2 Establishment and characterization of the GB-RHE model

**Fig. 5:**
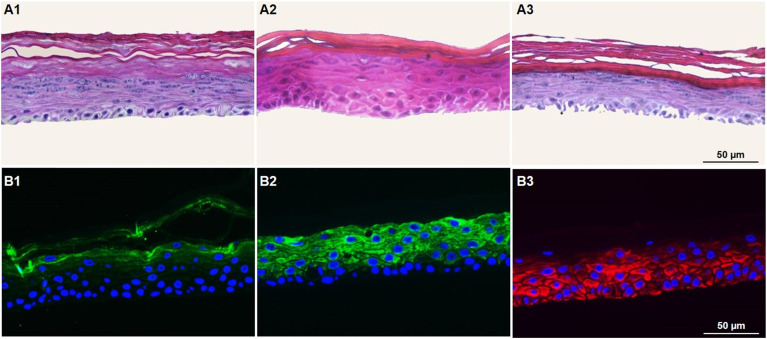
Optimization of the GB-RHE model. (A1) Histology of skin with 8 days of differentiation, (A2) skin with 9 days of differentiation, and (A3) skin with 11 days of differentiation. Immunohistochemistry analysis of skin developed at 9 days of differentiation, (B1) filaggrin, (B2) keratin 10 and (B3) keratin 14.

It is well known that the culture time of RHE models at the air–liquid interface, along with media composition, significantly influences the balance between cell proliferation and differentiation. An imbalance between these aspects can lead to tissues that either lack proper barrier formation or are fully differentiated. Thus, the next step was to optimize the GB-RHE protocol regarding the number of differentiation days to achieve a functional stratum corneum capable of protecting the living layers of the skin. Fig. 5 presents histological images of the GB-RHE with different differentiation times. As can be seen, 8 days GB-RHE present an average thickness of 81.25 ± 3.7 μm (A1), 9 days of 79.21 ± 0.8 μm (A2), and 11 days with an average thickness of 60.4 ± 5.0 μm (A3). The newly developed GB-RHE comprises distinct layers comparable to the human epidermis. The skin constructed with 9 days of differentiation exhibited a well-defined stratum corneum, resulting in a higher OD (0.95) compared to the 8 days (0.8) and 11 days (0.7). It also achieved a transepithelial electrical resistance (TEER) of 1649 ohms cm^2^. The GB-RHE model reveals distinct layers, including the stratum corneum, stratum granulosum, stratum spinosum, and stratum basal, discernible through the expression of specific markers filaggrin, keratin 10, and keratin 14 as illustrated in Fig. 5B. Filaggrin, situated in the stratum corneum, contrasts with keratin 10 found in the suprabasal layers, while keratin 14 is localized within the cells of the basal layer. Additionally, as outlined in OECD TG 439, morphology is a primary criterion for quality control and model acceptance. The results confirm that the GB-RHE model meets the required morphological specifications. Consequently, the pre-validation tests and irritation assessments of NPs were performed using the GB-RHE established with a 9 days air–liquid differentiation period.

#### 3.2.1 OECD TG 439 to assess chemical irritation

**Fig. 6:**
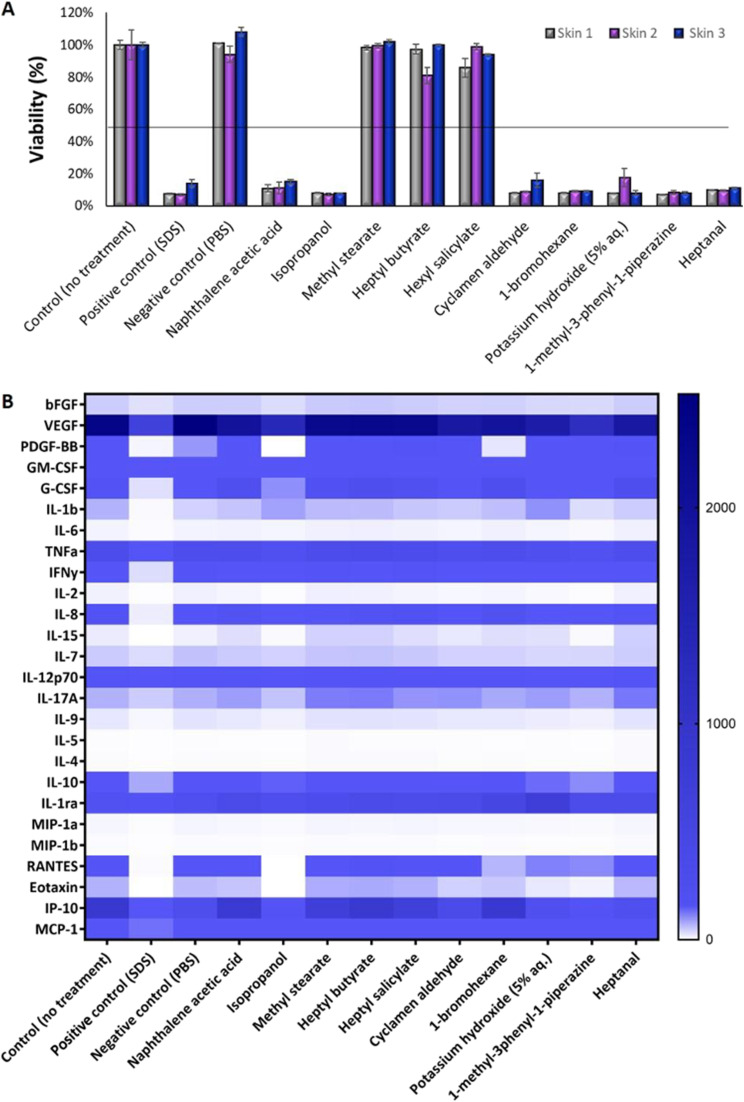
Dermal irritation test following OECD TG 439. (A) A triplicate of independent experiments is called skin 1, skin 2, and skin 3. (B) The rainbow graph demonstrates different cytokine secretion (pg mL^−1^) after exposure to the 10 chemicals.

The viability of the GB-RHE model was assessed using various controls: untreated cells, SDS as a positive control, and PBS as a negative control. After a 42 minutes exposure and 48 hours incubation period, these samples underwent the MTT assay, the standard OECD TG 439 method for evaluating cell viability in RHE models. Considering that being within the lower limit (viability <50%) for the positive control and upper limit (viability ≥50%) of acceptance for the untreated controls and negative control, the GB-RHE model can be used to evaluate adverse reactions of skin to non-irritating and irritating chemicals. This assay was conducted over three independent experiments, where skins 1 and 2 were produced at INMETRO's facilities and skin 3 at Grupo Boticário's facilities. As shown in Fig. 6A, 8 of the 10 reference substances from the OECD TG 439 were correctly classified, corresponding to a specificity of 60% and a sensitivity of 100%. The irritation test results of the three independent experiments were consistent across all chemical substances tested, including the 2 substances that behaved as false positives (naphthalene acetic acid and isopropanol).

The tested substances were categorized based on their impact on the reconstituted human epidermis's (RHE) viability according to the OECD TG 439 (Fig. 6A). Substances with cell viability above the 50% threshold, such as isopropanol, methyl stearate, heptyl butyrate, and hexyl salicylate, were classified as non-irritants. In contrast, substances such as cyclamen aldehyde, 1-bromohexane, potassium hydroxide (5% aq.), and heptanal, which exhibited cell viability below 50%, were identified as irritants. Notably, naphthalene acetic acid and isopropanol also fell into the irritant category, displaying lower viability.

Fig. 6B depicts a heatmap analysis revealing distinct cytokine expression patterns in response to various tested substances. The control group, which consisted of untreated skin, and the negative control group (PBS) both exhibited a similar cytokine profile, displaying minimal pro-inflammatory cytokine secretion, thereby providing a reliable baseline for low inflammatory activity. Conversely, the positive control group exposed to sodium dodecyl sulfate (SDS) treatment demonstrated a robust pro-inflammatory response characterized by a significant increase in pro-inflammatory cytokines (IL-1b, IL-6, TNFa, IFNγ, IL-2, IL-8, and IL-17A). This outcome validated the assay's sensitivity in detecting irritants that trigger an inflammatory process.

Non-irritant compounds, including methyl stearate, heptyl butyrate, hexyl salicylate, and even isopropanol, which is typically a non-irritating substance but was shown to be irritant in this test, consistently exhibited low levels of pro-inflammatory cytokines, in alignment with their classification as non-irritants. Irritant compounds, such as cyclamen aldehyde, 1-bromohexane, potassium hydroxide (5% aq.), 1-methyl-3-phenyl-1-piperazine, and heptanal, induced a robust pro-inflammatory response, marked by elevated levels of pro-inflammatory cytokine, thus emphasizing their potential to irritate the skin. Interestingly, naphthalene acetic acid, classified according to the OECD as non-irritating (Table 1) and in our study shown to be irritating, provoked a moderate pro-inflammatory response, defying its conventional categorization for BG-RHE.

Table 2 presents a comparison of the GB-RHE model against other reference methods validated in OECD TG 439, focusing on viability in response to chemicals and predictive capacity. Except for naphthalene acetic acid and isopropanol, the GB-RHE model showed similar results to the other reference models (Table 2A), achieving a sensitivity of 100%, specificity of 60% and accuracy of 80% (Table 2B).

GB-RHE model compared to other reference methods validated in OECD TG 439. (A) Viability in response to chemicals. (B) Predictive capacity. These data are from the original validation study report (OECD, 2021)^27^

**Table:** 

A				
Test substance	EpiSkin™ (SM)	EPiDerm™ SIT (EPI-2000)	SkinEthic™ RHE	GB-RHE
**Non-irritating substances (no cat.)**				
Naphthalene acetic acid	92.3 ± 5.2	100.7 ± 8.4	104.0 ± 12.9	12.3 ± 0.2
Isopropanol	88.1 ± 8.7	65.6 ± 16.5	101.0 ± 11.3	7.6 ± 0.0
Methyl stearate	98.5 ± 11.3	107.7 ± 4.9	104.4 ± 15.8	100.0 ± 0.1
Heptyl butyrate	102.0 ± 4.2	104.1 ± 4.2	92.1 ± 17.5	92.8 ± 0.1
Hexyl salicylate	89.0 ± 1.8	106.9 ± 5.2	95.9 ± 12.5	92.9 ± 0.1

**Irritant substances (cat. 2)**				
Cyclamen aldehyde	25.4 ± 12.1	18.5 ± 16.2	1.7 ± 0.9	11.0 ± 0.1
1-Bromohexane	24.4 ± 15.9	16.9 ± 2.5	1.3 ± 3.9	8.7 ± 0.0
Potassium hydroxide (5% aq.)	9.3 ± 10.0	4.3 ± 1.0	16.7 ± 17.0	11.1 ± 0.1
1-Methyl-3-phenyl-1-piperazine	23.8 ± 17.8	7.3 ± 2.7	8.2 ± 7.1	7.7 ± 0.0
Heptanal	16.6 ± 13.6	5.1 ± 0.3	1.3 ± 0.9	10.1 ± 0.0

**Table:** 

B					
	OECD TG 439 (2021) (%)	EpiSkin™ (SM) (%)	EPiDerm™ SIT (EPI-2000) (%)	SkinEthic™ RHE (%)	GB-RHE (%)
Sensitivity	≥80	80	80	90	100
Specificity	≥70	70	70	80	60
Accuracy	≥75	75	80	85	80

### 3.3 Effect of titanium dioxide nanoparticles on the GB-RHE model

#### 3.3.1 Physicochemical characterization of titanium dioxide nanoparticles

**Fig. 7:**
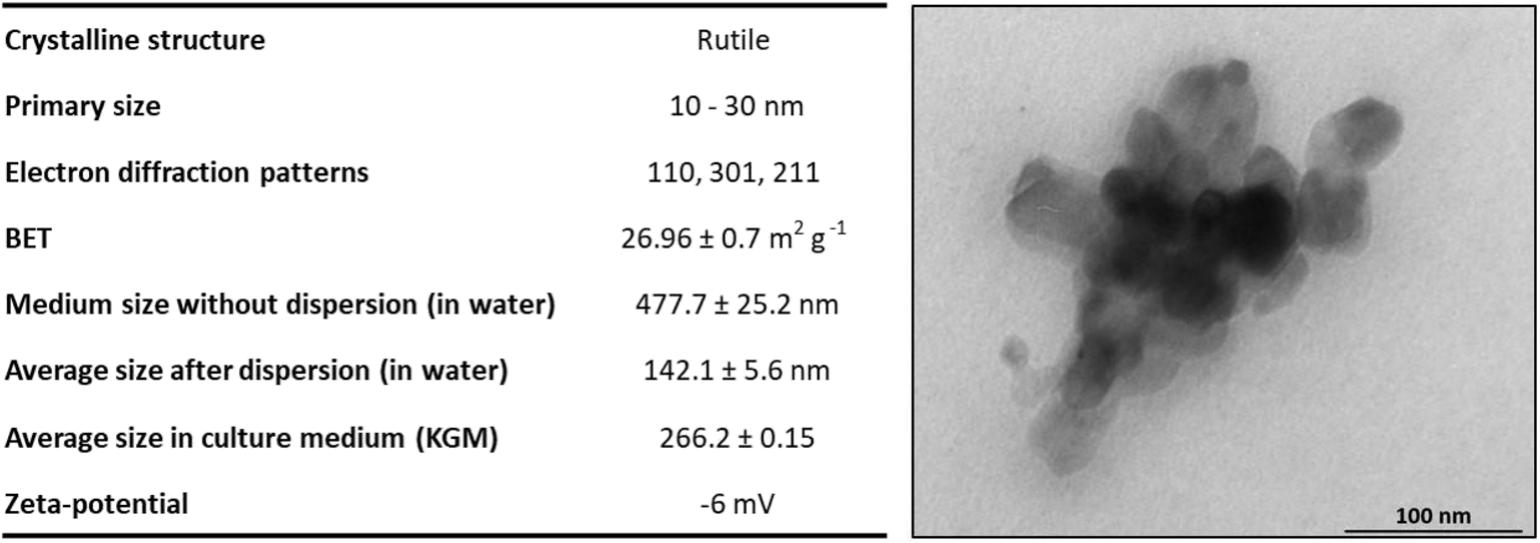
Table summarizing the physicochemical characterization of TiO_2_ NPs. The hydrodynamic diameter of TiO_2_ NPs was characterized before and after implementing a dispersion protocol using a probe sonicator. Transmission electron microscopy images of a cluster of TiO_2_ NPs in KGM medium culture. Adapted from Sanches *et al.* 2019.^28^

Commercially available rutile NPs were used to mimic the TiO_2_ NPs present in sunscreens. These rutile NPs have a primary size of 10 to 30 nm. As already described,^28^ it was observed by dynamic light scattering (DLS) that after contact with distilled water, these NPs agglomerate and had an average hydrodynamic size of 477.7 ± 25.2 nm, being necessary to disperse them using a probe sonicator. The physicochemical characterization of these NPs and the dispersion protocols in water and cell culture media can be found in detail in Sanches *et al.* 2019.^28^Fig. 7 summarizes the physicochemical characteristics of NPs before and after dispersion. The average hydrodynamic size of TiO_2_ NPs was 142.1 ± 5.6 nm after dispersion in water and 266.2 ± 0.15 nm upon incubation in a KGM culture medium. TEM micrographs confirm the result observed by DLS.

#### 3.3.2 Assessment of TiO_2_ NP irritation employing OECD TG 439 and cytotoxicity

The next step of this work was to evaluate whether TiO_2_ NPs are classified as irritating or cytotoxic in the GB-RHE model. Literature reports suggest that TiO_2_ NPs can interfere with the MTT assay in studies involving cell monolayers since they may remain adhered after washing and direct contact with the MTT reagent. However, this interference is not observed in three-dimensional models since NPs are added on the skin's surface (on top of the insert) and washed off before MTT is placed at the bottom of the well. To ensure no interference occurred, we adapted the protocol “DB-ALM Protocol #135: SkinEthicTM Skin Irritation Test”, conducting tests with batches of both dead and live GB-RHE.

**Fig. 8:**
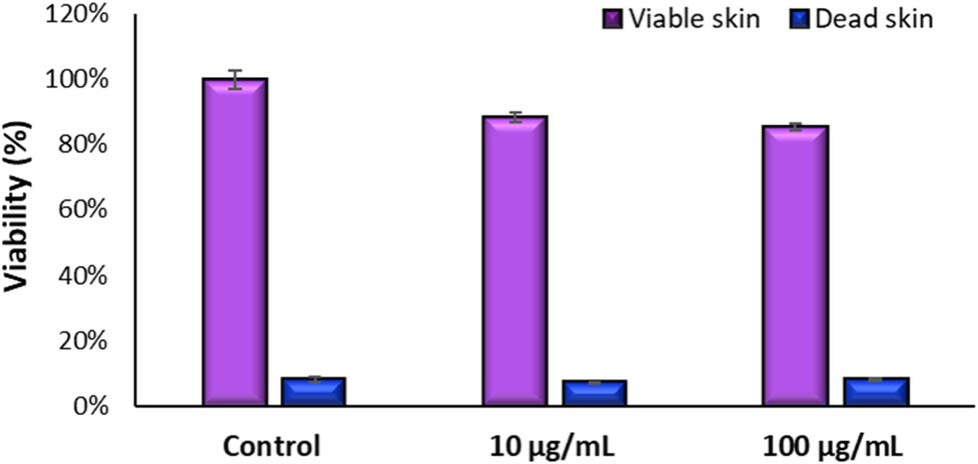
Interference test of MTT assay with TiO_2_ NP using dead and live GB-RHE skins.

Fig. 8 demonstrates the high viability of the model exposed to TiO_2_ nanoparticles on living skin, maintaining viability above 50% for both concentrations tested. On the other hand, dead skin shows low viability in all scenarios, including controls and NP-exposed samples, indicating that TiO_2_ NPs do not interfere with the MTT assay performed in the skin models. Had there been interference from TiO_2_ NPs, we would expect to see variability in viability between the dead skin exposed to the NPs and their respective controls.

**Fig. 9:**
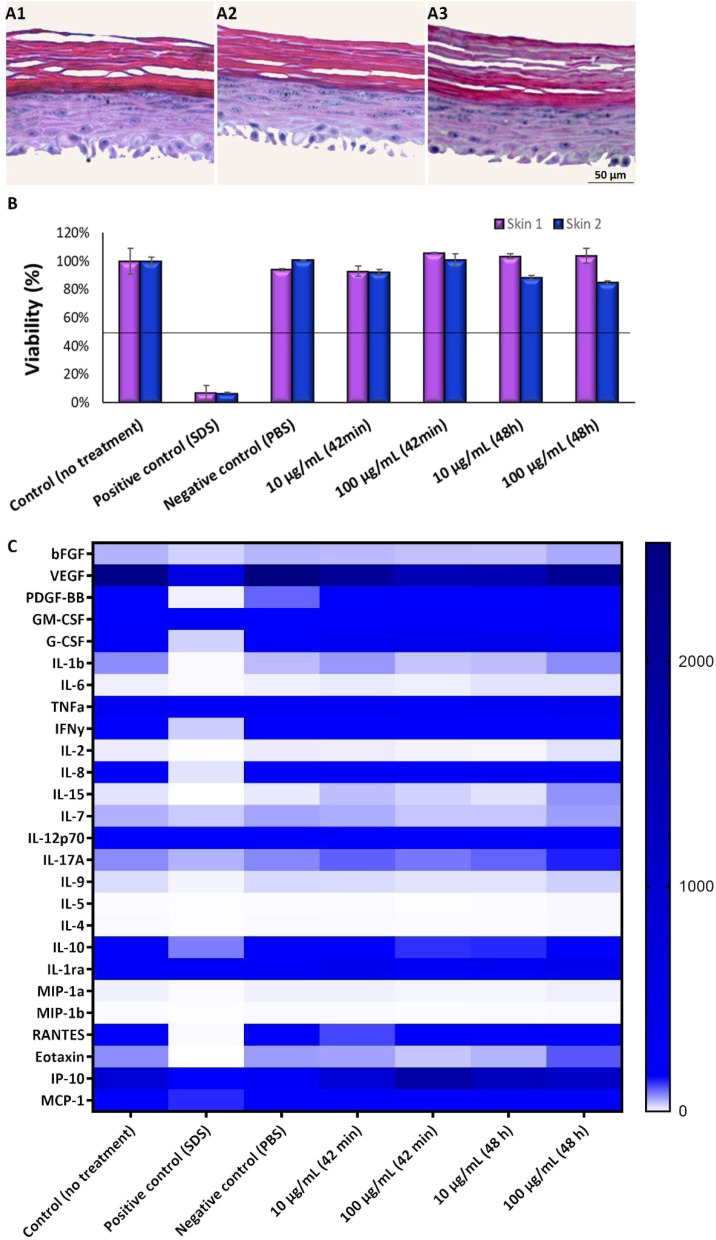
Assessment of irritation and cytotoxicity of TiO_2_ NPs. Histology of skin with 9 days of differentiation exposed to different concentrations of TiO_2_ NPs, (A1) control (0 μg mL^−1^), (A2) 10 μg mL^−1^, (A3) 100 μg mL^−1^. (B) TiO_2_ NP irritation test (42 minutes of exposure) and cytotoxicity (48 hours of exposure). Duplicate of independent experiments, called skin 1 and skin 2. (C) Rainbow graph demonstrates different secretion of cytokines (pg mL^−1^) after exposure to different concentrations of TiO_2_ NPs.

Fig. 9 shows the result of the test of irritation and toxicity of TiO_2_ NPs. To determine the irritation potential of TiO_2_ nanoparticles in the GB-RHE skin model, a 42 minutes exposure was employed, mirroring the standard duration for chemical dermal irritation in these studies, which used the SkinEthic RHE™ model. For cytotoxicity evaluation, the exposure period was extended to 48 hours. Results demonstrated that TiO_2_ NPs were considered non-irritant and non-cytotoxic, even for the highest concentration of NPs studied (100 μg mL^−1^).

As keratinocytes are the predominant cell type in the epidermis and play a crucial role in the skin's immune response, cytokine quantification was analyzed in the supernatant of the RHE model upon TiO_2_ NP exposure, providing insight into the immune and inflammatory responses induced by these nanoparticles. Results show an increase in IL-6, IL-15, IL-7, IL-17A, IL-9, and eotaxin release upon TiO_2_ NP exposure at the highest concentration tested and with an exposure time of 48 hours.

#### 3.3.3 Internalization of TiO_2_ NPs on GB-RHE model

**Fig. 10:**
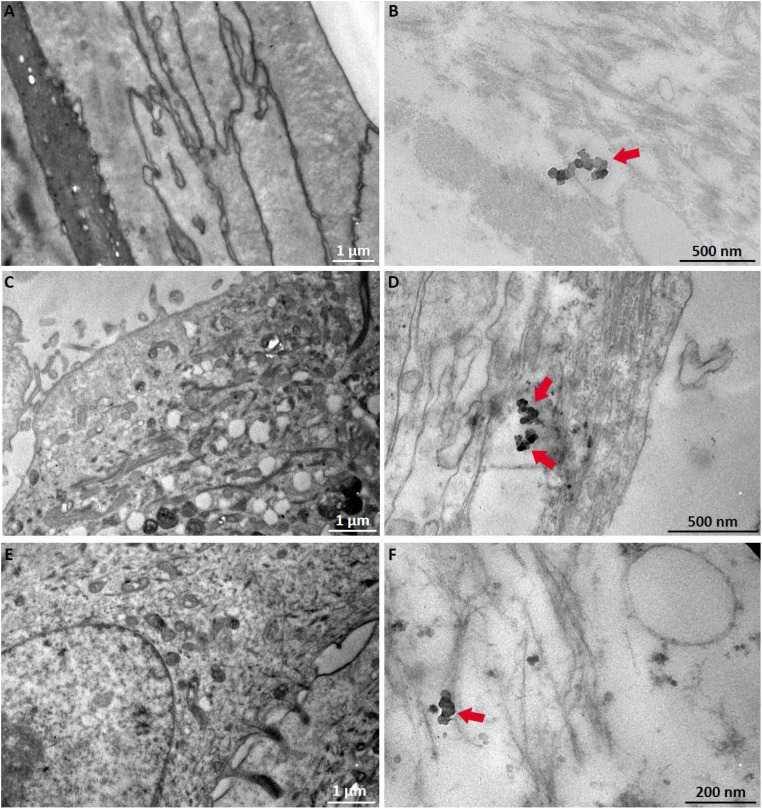
TEM micrographs of the GB-RHE skin model from the control (A), (C) and (E) and after 48 hours of TiO_2_ exposure (B), (D) and (F). The black arrows indicate TiO_2_ NPs.

Transmission electron microscopy evaluated whether the TiO_2_ NPs can cross the stratum corneum and internalize into the viable cell layers. Fig. 10 shows micrographs of cross-sections of the GB-RHE model exposed (B), (D) and (F) or not (A), (C) and (E) to TiO_2_ NPs for 48 hours. Transmission electron microscopy revealed microstructures of the human epidermis. It was possible to observe TiO_2_ NPs in contact with what seems like keratin filaments, confirming that the NPs can internalize in the stratum corneum (Fig. 10B, D and F). However, additional studies will be necessary to identify whether these NPs can reach the epidermis's basal layers or are retained in the more superficial layers after internalization.

## 4. Discussion

Biological tissues exhibit complex heterogeneity in terms of cellular composition and differentiation stages. This diversity ranges from tissue stem cells, proliferative and undifferentiated intermediate types, to differentiated and terminal cells.^29^ As a result, primary cultures of these tissues are inherently heterogeneous.^29,30^ Therefore, the methodology used *in vitro* cultivation must consider, among other things, a deep understanding of the cell type of interest, the monitoring of morphological and proliferative aspects, and the assessment of viability and proliferation rate, which must be carried out at the time of cultivation. Primary cultures are sensitive to *in vitro* conditions and can quickly change response to different factors, resulting in alterations of cellular properties, metabolism, and protein expression,^1,31^ which can lead to premature cell senescence.^32^

Keratinocytes start as small, young, proliferative cells with diameters ranging from 5 to 20 μm, typically fusiform or cuboidal in shape. As they differentiate, they expand in size, reaching up to about 100 μm, and transition from a fusiform shape to a more circular and irregular form. Sometimes, these cells may become multinucleated during this maturation process.^33^ It was observed that the success of the RHE constructs depended on the use of primary cultures of proliferative and young keratinocytes, especially when the cultures maintained the proliferation in the form of a colony with very regular borders (holoclone). In the early 1990s, Pellegrini discussed the need to use young cells keratinocytes with high proliferative capacity for the success of cell therapies. He characterized the cellular characteristic aspects and called them holoclones,^34,35^ which remain viable and proliferative over long periods, leading to the success of procedures that use these cells. In this article, we observed that keratinocyte proliferation exhibited exponential growth up until the third passage, after which there was a tendency for the proliferation rate to decrease. These findings underscore the importance of monitoring the cellular state as a crucial factor in reducing the inherent variability of the RHE. Once controlled, the reproducibility of the RHE model becomes an advantage compared to the animal model.^4^ Standardization of cell isolation and monitoring of the batch quality is essential to ensure the effective construction of the RHE model.

The successful collection of primary cultures and their proliferative properties are inextricably linked to the characteristics of the biological tissue donor, such as age and ethnicity. Primary cultures of normal cells derived from younger individuals exhibit higher proliferative capacity and lower expression of phenomena associated with senescence and apoptotic markers. Although effective techniques for cultivating keratinocytes have been outlined and widely employed for decades, a limited number of cells per tissue exists, and there is significant room for enhancement, refinement, and a deeper comprehension of *in vitro* epidermal models.

According to the histological results, we observed that the average thickness of the GB-RHE model was 76.25 μm comparable to human breast thickness (76.9 ± 26.2 μm)^36^ and presented well-differentiated layers of stratum basale, stratum spinosum, stratum granulosum, and multilayered functional stratified stratum corneum.^10^ A similar thickness and morphology were obtained by comparing GB-RHE with the SkinEthic™ model.^27,37^ The barrier integrity of the GB-RHE model presented 1649 ohms cm^2^ of TEER. This value indicates a robust epidermal barrier, as already demonstrated in other widely used OECD-validated models. For comparison, the EpiDerm™ model typically reports TEER values of 500 ohms cm^2^ in standard conditions and other models without formal regulatory acceptance, such as Keraskin™ >500 Ω cm^2^ and dermo-epidermal skin equivalents >1000 Ω cm^2^. The higher TEER value observed in the GB-RHE model suggests a stronger barrier function. This robust barrier function makes the GB-RHE model suitable for assessing the skin irritation potential of both chemical irritants and nanoparticles, reinforcing its reliability and applicability in line with OECD TG 439.

The GB-RHE model met the OECD minimum acceptance criteria: sensitivity ≥80%. The irritation results reflect individual variability concerning chemical substances in the population. Two of the 10 chemical substances (naphthalene acetic acid and isopropanol) were incorrectly classified in all replicates, behaving as false positives, and 8 of the 10 substances were classified correctly, which resulted in a specificity of 60% and sensitivity of 100% across batches. Pedrosa *et al.* demonstrated that, in one of three replicates of independent experiments, naphthalene acetic acid produced a viability value below 50%, indicating variability in its classification as an irritant.^38^ Regarding isopropanol, its high permeability might have allowed it to pass through the sides of the inserts, reaching the basal layers of the skin, which could have contributed to the decreased viability observed in the GB-RHE model. Additionally, it's important to consider that there may be ethnological differences in tolerance to specific chemicals; however, these assumptions require further investigation to be substantiated. The transfer phase between Grupo Boticário and Inmetro confirmed that the standard operating procedures for producing the GB-RHE model and performing skin irritation tests were successfully implemented and replicated, demonstrating inter-laboratory reproducibility. It is important to emphasize that full validation requires testing all 20 reference substances outlined in the performance standards of TG 439, along with additional assessments of quality control, reproducibility, and barrier function. Efforts are currently underway to thoroughly validate the GB-RHE model to enhance the assessment of its predictability, sensitivity, and specificity.

Analyzing the pro-inflammatory cytokine profile in response to chemical exposure is essential for toxicological risk assessment and gaining a deeper understanding of the substance's mode of action, particularly within the OECD TG 439 models for skin irritation. Our findings show that irritant chemicals such as cyclamen aldehyde and heptanal significantly upregulate cytokines like IL-1β and TNFα, which are critical to inflammation and immune response.^39,40^ This aligns with their classification as irritants and their liquid state, which likely facilitates skin penetration and the subsequent inflammatory response.^41^ In contrast, non-irritants, exhibited minimal cytokine activity, corroborating their potential to remain non-reactive in terms of cutaneous inflammation. Correlating these cytokine signatures with cell viability data offers a dual approach for evaluating chemical sensitizers, supporting the ongoing shift towards animal-free testing methods.

The alignment of our cytokine data with traditional irritancy categories highlights the effectiveness of the GB-RHE model in differentiating between irritants and non-irritants. This supports its potential adoption in regulatory toxicology as a predictive tool for assessing irritation potential and the safety of consumer products, ultimately aiding in the protection of human health.^42^ Thus, cytokine profiling in the RHE model emerges as a critical parameter for understanding the mechanisms of skin irritation, reinforcing the relevance of OECD TG 439 for chemical risk assessment.^43^

Several studies of skin corrosion and irritation of NPs employ two-dimensional *in vitro* testing with skin-derived cell lines. However, data using equivalent human skin models are urgently needed to enable tests under more realistic scenarios. Currently, no standardized *in vitro* method exists for the toxicological evaluation of NPs. This work primarily aimed to determine whether existing protocols for conventional chemical substances, such as those outlined in OECD TG 439, could effectively assess the irritation potential of NPs. Unique physicochemical characteristics of NPs—such as size, surface reactivity, and aggregation behavior—may influence their interaction with skin tissues and are not well captured by traditional techniques. By addressing these aspects, we aim to expand preclinical safety assessment methods for NPs without relying on animal testing.

This study represents a step toward bridging this gap, demonstrating the potential GB-RHE, in tackling the unique challenges posed by NPs within a regulatory framework. The present article evaluates the GB-RHE model's performance in assessing the irritation potential, following OECD TG 439 and its capability to evaluate internalization and the hazards associated with TiO_2_ NPs exposure. Our results demonstrated that TiO_2_ NPs were non-irritating and non-toxic. However, high-resolution techniques revealed that TiO_2_ NPs were internalized throughout the epidermal layer. This finding might be linked with the impaired barrier properties of the GB-RHE model that already demonstrated higher sensitivity to non-irritant substances.^44,45^

The literature regarding the dermal penetration potential of TiO_2_ NPs from sunscreens exhibits controversial results. While several articles describe the opposite, the scientific community demonstrates the penetration of TiO_2_ NPs in healthy but also damaged or lesioned skin.^1,23,28^ This study highlights the great potential of RHE models in evaluating irritation, toxicity, and cellular internalization of TiO_2_ NPs found in cosmetics. Several features of NPs including shape, size, surface functional groups, and hydrophilicity, elicit spatiotemporal responses to the interactions with cellular components. However, the magnitude and the result of such interactions differ between NPs and cell models.

The effects of NPs on cytokines secretion by keratinocytes can vary depending on several factors, including the type, size, surface characteristics, concentration, and duration of exposure to NPs.

Exposure of the GB-RHE model to TiO_2_ NPs resulted in an increased secretion of IL-6, a pro-inflammatory cytokine involved in immune responses and inflammation. Additionally, interleukin-15 (IL-15) was secreted at higher levels than the control. IL-15 is a pro-inflammatory cytokine that plays a crucial role in the immune system and regulates immune responses, inflammation, and activating different immune cells, such as natural killer and memory CD8+ T cells. These cells are important for immune surveillance and defense mechanisms in the skin. Interestingly, tissue damage or stress conditions, including NP exposure, can also lead to IL-15 secretion.

IL-17 is a cytokine crucial for T cell development and maintenance, although the mechanisms triggering its release in keratinocytes are less studied.^39,40^ The release of interleukin-17A (IL-17A) by keratinocytes in response to various stimuli, including NPs, has been studied in the context of skin inflammation and immune responses. IL-17A is a pro-inflammatory cytokine that plays a role in the recruitment and activation of immune cells, and it is associated with inflammatory conditions such as psoriasis. Research suggests that NPs exposure can induce the release of IL-17A from keratinocytes, mediated through specific signalling pathways involved in inflammation.^46^ The release of IL-17A may contribute to the recruitment of immune cells and the development of an inflammatory microenvironment. The presence of IL-17 in response to TiO_2_ NPs indicates a potential for enhanced immune activation, which might not manifest as immediate irritation but could contribute to long-term inflammation.

IL-9 is a cytokine commonly associated with T-helper 9 cells and plays a role in allergic and inflammatory responses. The release of IL-9 in the GB-RHE model suggests that, although TiO_2_ NPs do not cause acute irritation, they may have the potential to sensitize the immune system, possibly leading to allergic reactions upon repeated exposure. This possibility is further supported by elevated levels of eotaxin, a chemokine responsible for recruiting eosinophils. Eosinophils are typically involved in allergic inflammation and increased levels of eotaxin suggest that TiO_2_ NPs might provoke a Th2-type immune response, indicating a risk for allergic sensitization even in the absence of acute irritation. Various factors, including inflammatory stimuli, allergens, and other environmental triggers, can influence eotaxin release from keratinocytes.

In summary, we observed an inflammatory response by the increased secretion of several pro-inflammatory cytokines and chemokines. Skin inflammation is known to be one of the alterations associated with NPs exposure, with epidemiological studies demonstrating the progression of inflammatory skin diseases such as atopic dermatitis. Inflammation serves as an early immunological response to exogenous nanomaterials. Keratinocytes can produce pro-inflammatory cytokines, such as IL-8, IL-6, TNF-α, and IL-1β, which play crucial roles in skin tissue's inflammatory and immunologic reactions to irritants.^46^ Research in nanotoxicology has investigated the impact of various NPs, including TiO_2_ NPs, silver, zinc oxide, and others, on keratinocytes and cytokine secretion. It is known that NPs can induce an inflammatory response, leading to the release of inflammatory cytokines such as interleukin-1 beta (IL-1β), interleukin-6 (IL-6), and tumor necrosis factor-alpha (TNF-α).^45^

Similar divergent cytokine responses have been reported for other irritants and NPs, highlighting the necessity for multi-parameter evaluations when assessing NPs safety. These results emphasize the importance of considering both acute irritation and longer-term immune effects in NPs safety assessments. The data underscores the need to evaluate acute viability, chronic exposure scenarios, and cytokine-mediated immune responses for a comprehensive understanding of nanoparticle safety.

Although significant progress has been made in the GB-RHE pre-validation, further steps should include conducting an inter-laboratory validation study across various scenarios to demonstrate its robustness and reliability. This approach aligns with conventional regulatory validation processes, thereby enhancing the model's legitimacy and encouraging its widespread adoption.

## 5. Conclusions

In this study, the GB-RHE model was pre-validated through analyses of structural morphology, cell viability, barrier function, and inflammatory response. TEER measurements confirmed its robust epidermal barrier, comparable to other OECD-validated models, and the model demonstrated high sensitivity (100%) and acceptable specificity (60%) when tested with a subset of reference substances from OECD TG 439. These results support its potential as an alternative *in vitro* method for skin irritation testing. Full validation with all 20 reference substances remains a priority to further establish the model's reliability and predictive capacity.

The GB-RHE model effectively assessed the irritant potential of TiO_2_ nanoparticles (NPs), demonstrating their classification as non-irritant under the worldwide harmonized classification system. While TiO_2_ NPs did not interfere with the MTT assay or penetrate deeper layers of the epidermis, elevated cytokine levels, including IL-6 and IL-17A, suggest possible immune modulation and a predisposition to chronic inflammation or allergic sensitization under prolonged exposure. These findings highlight the need to integrate cytokine profiling into nanoparticle safety assessments.

The GB-RHE model emerges as a promising tool for replacing animal testing in skin irritation studies and advancing nanoparticle toxicology. Future efforts should focus on completing validation, improving reproducibility, and expanding its regulatory applicability. Collaboration across institutions and the adoption of standardized protocols will be essential for broader implementation and accessibility, enabling the model's application in dermatology, cosmetology, and toxicology.

## Data availability

The raw quantitative data supporting the findings of this study, including data used to generate Fig. 3, 6, 8 and 9, have been deposited in the Open Science Framework (OSF) repository. These datasets are publicly accessible and can be retrieved *via* the following DOI: https://doi.org/10.17605/OSF.IO/B42UZ. The deposited data include detailed numerical values and experimental outcomes from the OECD 439 test guideline employed to evaluate the skin irritation potential of titanium dioxide nanoparticles in a skin equivalent model. This registration ensures compliance with standards of transparency, reproducibility, and data sharing, facilitating the reuse of findings by the scientific community.

## Author contributions

Conceived and designed the experiments: A. R. R., J. M. G., P. L. S., C. M. C., G. G. A., A. P. M., D. C. S. The experiments were carried out by: P. L. S., C. M. C., R. B. V. C., G. G. A., B. B., M. C. C. C. Analyzed the data: P. L. S., A. R. R., J. M. G., C. M. C., R. B. V. C., G. G. A. Wrote the article: P. L. S., A. R. R. All authors reviewed the manuscript.

## Conflicts of interest

There are no conflicts to declare.
